# Successful Treatment with Selpercatinib for Ectopic Cushing’s Syndrome Due to Medullary Thyroid Cancer

**DOI:** 10.3390/curroncol29050282

**Published:** 2022-05-12

**Authors:** Oskar Ragnarsson, Marta Piasecka, Andreas Hallqvist

**Affiliations:** 1 Department of Endocrinology, Sahlgrenska University Hospital, SE-41302 Gothenburg, Sweden; marta.piasecka@vgregion.se; 2 Department of Oncology, Sahlgrenska University Hospital, SE-41302 Gothenburg, Sweden; andreas.hallqvist@vgregion.se

**Keywords:** medullary thyroid cancer, ectopic Cushing’s syndrome, paraneoplastic syndrome, hypercortisolism

## Abstract

Selpercatinib, a RET kinase inhibitor, is an effective treatment for patients with medullary thyroid cancer with RET mutations. In this paper, we present the case of a 62-year-old man with ectopic Cushing’s syndrome due to medullary thyroid cancer who received treatment with selpercatinib. Six months later, all the cushingoid features had resolved, and s-calcitonin had decreased from 580 pmol/L to 3.5 pmol/L (normal < 3). After further 6 months, s-calcitonin had normalized (1.5 pmol/L), and radiological evaluation showed a profound tumour volume reduction. We are aware of two other cases where treatment with selpercatinib has also been successful. Thus, selpercatinib may be a promising treatment alternative in patients with ectopic Cushing’s syndrome due to medullary thyroid cancer, especially when other treatment options are ineffective or not tolerated.

## 1. Introduction

Medullary thyroid cancer is a rare neuroendocrine tumour that accounts for approximately 1% of all thyroid cancers [1]. Medullary thyroid cancer can be either sporadic (60–75%) or hereditary [2,3]. The most common hereditary forms are multiple endocrine neoplasia type 2 A and B and familial medullary thyroid cancer syndrome, all autosomal dominant disorders [2,3].

In rare cases, patients with medullary thyroid cancer may develop excessive cortisol production due to ACTH production from the tumour, a paraneoplastic state called ectopic Cushing’s syndrome. Ectopic Cushing’s syndrome is a serious disorder associated with a very poor prognosis if it is not promptly diagnosed and treated [4].

## 2. Case Presentation

In August 2020, a 62-year-old man was referred to the department of endocrinology at our hospital due to severe muscle weakness that had gradually developed over the past 6 months. On clinical examination, the patient had typical Cushingoid features, including central obesity with supraclavicular and dorsocervical fat accumulation, a round face, proximal muscle atrophy and weakness, facial plethora, therapy-resistant hypertension, and hypokalemia. Endocrine work-up revealed ACTH-dependent Cushing’s syndrome: S-cortisol following 1 mg overnight dexamethasone suppression was 894 nmol/L (normal < 50), S-cortisol at midnight was 690 nmol/L (normal < 130), late-night salivary cortisol was 27 nmol/L (normal < 1), and ACTH was 25 pmol/L (normal 1.6–14). Seven years earlier, the patient was diagnosed with medullary thyroid cancer with liver and lymph node metastasis. Following thyroidectomy, liver resection and surgical removal of mediastinal lymph nodes, s-calcitonin decreased from 5500 pmol/L to 150 pmol/L (normal < 3). In the following years, due to the progression of lymph node metastasis, treatment with tyrosine kinase inhibitors was tested, first with vandetanib and then with cabozantinib. Vandetanib was discontinued after 10 months of treatment because of a lack of effectiveness, and cabozantinib was discontinued due to troublesome side effects, mainly abdominal pain and diarrhoea. Temozolomide, capecitabine and peptide receptor radionuclide therapy with lutetium were also tested but were ineffective. 

**Figure 1:**
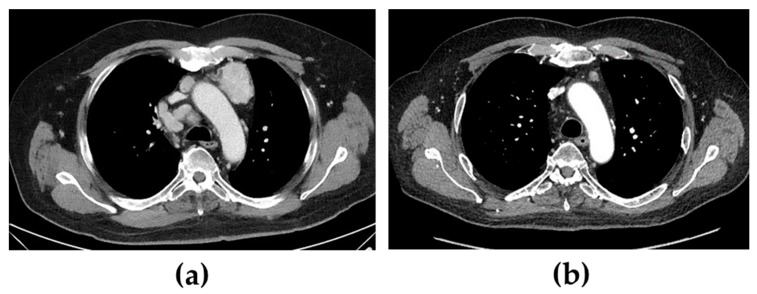

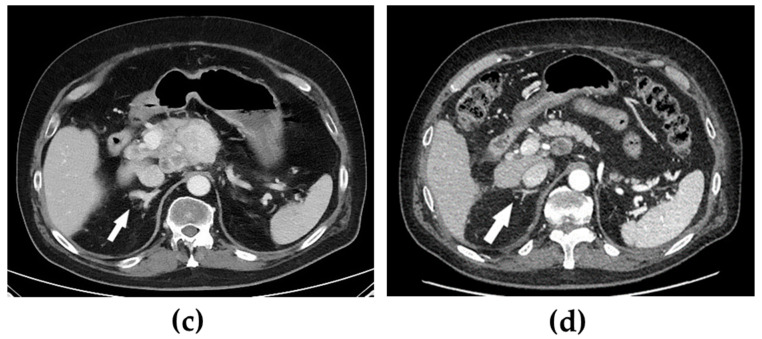
Computed tomography showing mediastinal and abdominal lymph node metastases before (**a**,**c**) and 12 months after (**b**,**d**) treatment with selpercatinib was started. Of additional note is a hyperplastic adrenal gland (arrows) before treatment that decreased in volume following treatment.

When the patient was diagnosed with Cushing’s syndrome, treatment with metyrapone (750 mg three times daily) was initiated. Two days later, s-cortisol was under the limit of detection, and hydrocortisone was added in a block-and-replacement regime. On a histopathological re-examination of the tumour, immunohistochemical staining for ACTH was positive. Four days after treatment with metyrapone was started, after an oncogenic mutation in exon 16 of the *RET* gene was confirmed, i.e., the most commonly affected exon in sporadic cases [5], treatment with selpercatinib (160 mg two times daily) was also initiated. At that time, the patient had several lymph node metastases, the largest being found in the mediastinum (47 × 35 mm) and between the pancreas and aorta (largest diameter was 69 mm) (Figure 1a,c). Six months later, all the cushingoid features had resolved, s-calcitonin had decreased from 580 pmol/L to 3.5 pmol/L (normal < 3), and the metastases had decreased significantly in size. 

After further 6 months, s-calcitonin had normalized (1.5 pmol/L), and radiological evaluation showed a profound tumour volume reduction (Figure 1b,d). Furthermore, due to the excellent biochemical and radiological response to selpercatinib, both metyrapone and hydrocortisone were discontinued without a subsequent recurrence of the hypercortisolism. In fact, the patient had instead developed adrenal insufficiency with both unstimulated and stimulated s-cortisol < 60 nmol/L, also indicating an excellent response to selpercatinib. Hydrocortisone replacement was therefore reintroduced.

At the most recent follow-up visit, after 18 months of treatment with selpercatinib 160 mg twice daily, the patient was still in remission and did not experience any significant side effects. In thoracic and abdominal computed tomography, no metastasis could be identified, s-calcitonin was normal, and glucocorticoid replacement therapy was still required for adrenal insufficiency. 

## 3. Discussion

Ectopic Cushing’s syndrome is a rare paraneoplastic disorder seen in approximately 1% of patients with medullary thyroid cancer [6]. The clinical characteristics and treatment of 11 patients with ectopic Cushing’s syndrome due to medullary thyroid cancer were recently described [7]. As in patients with ectopic Cushing’s syndrome due to other malignant tumours, muscle weakness and diabetes mellitus are more prevalent than in patients with Cushing’s syndrome of adrenal and pituitary origin, and typical Cushingoid features with central fat accumulation are often lacking [8]. It was also shown that bilateral adrenalectomy and medical cortisol-lowering treatment are effective in controlling hypercortisolism in these patients [7]. 

It is interesting that the patient developed Cushing’s syndrome 7 years after he was diagnosed with medullary thyroid cancer and that re-examination of the tumour that was removed 7 years earlier revealed positive immunohistochemical staining for ACTH. The long interval between the diagnosis of medullary thyroid cancer and the development of ectopic Cushing’s syndrome is, however, not unique, and intervals of 3 and 11 years, respectively, have previously been reported [9,10].

Patients with medullary thyroid cancer and ectopic Cushing’s syndrome have a worse prognosis compared to patients with medullary thyroid cancer but without hypercortisolism [6,7]. Similar results have been found in patients with ectopic Cushing’s syndrome due to other cancer forms, such as small-cell lung cancer [11]. One of the reasons is undoubtedly the negative effects of hypercortisolism, i.e., the overproduction of cortisol increases the risk of cardiovascular disease, thromboembolism, and sepsis [12]. In fact, patients with Cushing’s syndrome, irrespective of aetiology, have a more than doubled mortality risk compared to the background population [13,14]. It is therefore of great importance to detect patients with medullary thyroid cancer who develop ectopic Cushing’s syndrome and to start cortisol-lowering medical treatment without delay.

Cortisol-lowering treatment with metyrapone was initiated as soon as Cushing’s syndrome was confirmed in our patient. Metyrapone blocks several enzymes in the adrenal cortex, which subsequently results in decreased cortisol production. The treatment of our patient was effective. In fact, after two days on metyrapone, the cortisol concentrations were very low, and replacement therapy with hydrocortisone became necessary to prevent symptomatic adrenal insufficiency. Thus, the patient received a block-and-replacement regime where metyrapone was used to block cortisol production and hydrocortisone replacement to achieve normal cortisol concentrations.

**Figure 2:**
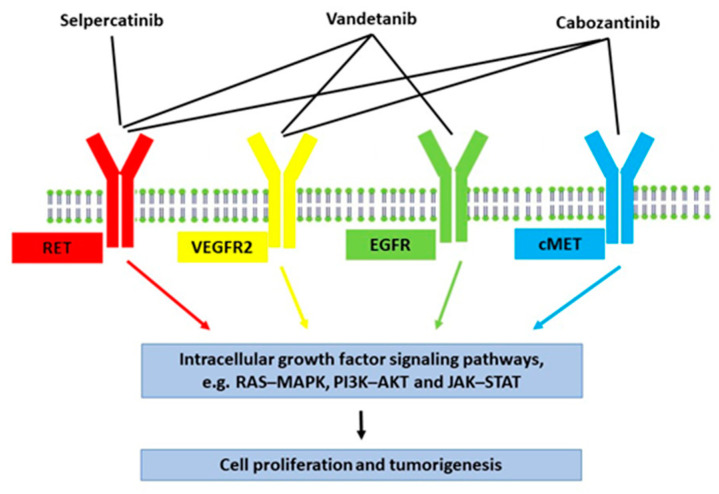
Schematic summary of the effects of tyrosine kinase inhibitors for metastatic medullary thyroid cancer. Vandetanib and cabozantinib are multi-targeted inhibitors. Vandetanib is mainly an inhibitor of vascular endothelial growth factor receptor-2 (VEGFR2), epidermal growth factor receptor (EGFR), and RET tyrosine kinase. Cabozantinib is mainly an inhibitor of VEGFR2, RET tyrosine kinase, and tyrosine-protein kinase Met (c-MET). Selpercatinib is a selective RET tyrosine kinase inhibitor, making it less toxic and less likely to cause side effects.

Several kinase inhibitors are available for the treatment of metastatic medullary thyroid cancer (Figure 2) [15]. Treatment with two of these, vandetanib and cabozantinib, were tested in our patient but were discontinued due to lack of effectiveness and side effects, respectively. In a study by Koehler et al., control over hypercortisolism was achieved in one patient with ectopic Cushing’s syndrome by using a tumour-targeted treatment with vandetanib and subsequently cabozantinib [7]. In another study, two patients with ectopic Cushing’s syndrome due to medullary thyroid cancer received vandetanib, but with inadequate control of the hypercortisolism, concomitant treatment with a cortisol-lowering agent was needed [16].

The majority of patients with medullary thyroid cancer have a mutation in the RET protooncogene, i.e., all patients with the hereditary forms and 56–65% of patients with sporadic disease [5,17]. Selpercatinib, a RET kinase inhibitor, is an effective treatment for patients with medullary thyroid cancer with RET mutations [18]. In a study by Koehler, one patient with ectopic Cushing’s syndrome due to medullary thyroid cancer received selpercatinib following bilateral adrenalectomy [7]. Thus, the effect on hypercortisolism could not be evaluated. In our case, selpercatinib had a remarkable effect on both tumour burden as well as hypercortisolism. In fact, when the cortisol-lowering therapy with metyrapone was discontinued, the cortisol concentrations remained low, illustrating that selpercatinib not only reduced the tumour volume and the concentrations of calcitonin but also reduced the production of ACTH, which in turn caused adrenal insufficiency, a well-known phenomenon in successfully treated patients with Cushing’s syndrome of all etiologies [19]. We are aware of two other cases where treatment with selpercatinib has also been successful [20,21]. 

## 4. Conclusions

Selpercatinib may be a promising treatment alternative in patients with ectopic Cushing’s syndrome due to medullary thyroid cancer, especially when other treatment options such as vandetanib and cabozantinib are ineffective or not tolerated.

## Data Availability

Raw clinical data can be obtained upon request from the corresponding author.
